# Morbidity, Treatment-Seeking Behaviour, and Out-of-Pocket Expenditures Among the Tribal Geriatric Population: A Cross-Sectional Study From a South Indian District

**DOI:** 10.7759/cureus.77546

**Published:** 2025-01-16

**Authors:** Ashish Solanki, Godavarti D Kumar, Godi R Varma, Bontha V Babu, Yadlapalli S Kusuma

**Affiliations:** 1 Income Tax Department, Ministry of Finance, Government of India, New Delhi, IND; 2 Community Medicine, All India Institute of Medical Sciences, New Delhi, New Delhi, IND; 3 Division of Socio-Behavioural, Health Systems, and Implementation Research, Indian Council of Medical Research, New Delhi, IND

**Keywords:** geriatric groups, india, indigenous communities, morbidity, out-of-pocket expenditures, scheduled tribes, treatment seeking

## Abstract

Objective: The objectives of the study are (i) to examine morbidity patterns, (ii) to explore the treatment-seeking behaviours, and (iii) to analyse the out-of-pocket expenditures (OOPEs) among Indigenous (referred to as tribal in India) elderly in the Visakhapatnam district of Andhra Pradesh, India.

Methods: A cross-sectional survey of 2,103 households was carried out. Data related to socio-demographic, health, and healthcare-seeking behaviours were collected for all the household members. There were 598 people aged 60 years and above in these households. Data on these 598 elderly people were analysed for the present study.

Results: The findings highlight the socioeconomic hardships of the tribal elderly, including reliance on labour-intensive work, low educational attainment, and high rates of widowhood. Data on the illnesses experienced in the past year revealed that 277 (46.3%) of the elderly experienced acute illnesses. Chronic conditions were present in 224 (37.5%) while 115 (19.2%) had musculoskeletal pain and 75 (12.5%) had hypertension. Multimorbidity was present in 29 (4.5%) of the elderly. A total of 226 (81.6%) sought care from government facilities for acute illnesses. For chronic illnesses, while 109 (48.7%) did not seek treatment, 88 (39.3%) relied on government healthcare facilities. Regarding hospitalisation, 12 (70.6%) were admitted in government facilities and five (29.4%) were admitted in private hospitals. The OOPEs were high when they sought care from private healthcare facilities. For acute illnesses, while the average (5% trimmed mean) OOPE was Indian rupee (INR) 254 when seeking care from government healthcare facilities, it was INR 4301 when seeking care from private facilities. For chronic illnesses, the expenditure for private healthcare (5% trimmed mean = INR 3403) was higher than when seeking care from government healthcare facilities (5% trimmed mean = INR 262). The average (5% trimmed mean) for hospitalisation was INR 6414 when admitted to government hospitals, and it was INR 46063 for private hospital admissions.

Conclusions: The illness burden is high among the tribal elderly. The OOPEs were high, particularly when seeking care from private healthcare facilities. The study emphasises the need to strengthen government healthcare services, particularly through health and wellness centres under India’s Ayushman Bharat programme. Access to government social support schemes such as old age pensions and health insurance should be improved. Health education and community partnerships are to be strengthened. Addressing these gaps is vital to alleviating health disparities among the tribal elderly and securing their access to India’s universal health coverage framework.

## Introduction

The geriatric population of India, aged 60 years and above, constitutes 8.3% of the total population [1,2] and is projected to increase to 13% by 2031 [3,4]. In the Indian state of Andhra Pradesh, around 8.3 million, i.e., 9.8% of the state’s population, are aged 60 years and above and is projected to increase to 16.4% by 2031 [3].

Indigenous communities in India, usually referred to as Scheduled Tribes (STs), represent approximately 8.6% of the nation's population [5]. These communities are characterised by distinct cultural practices, languages, and traditional lifestyles and often reside in geographically isolated regions. The Sustainable Development Goal 3 (SDG 3), i.e., “Ensure healthy lives and promote well-being for all ages”, includes indigenous people [6]. Indigenous people (referred to as tribes in India) are identified as a specific group in sustainable development goals. The present study was carried out in the Visakhapatnam district of Andhra Pradesh, an Indian state. Thirteen tribes, namely, Bagatha, Gadaba, Goudu, KondaKammara, Konda Dora, Khondh, Kotia, Mali, Nooka Dora, Porja, Valmiki, Dulia, and Mulia, including three particularly vulnerable tribes (PVTGs), namely, Gadaba, Khondh, and Porja, inhabit this area, constituting 14.4% of the district's population, which is 4.29 million according to the Census of India 2011 [1].

Indigenous people usually had poorer health outcomes globally [7]. The tribal population in India has poor health [8]. The health indicators among the Indian tribes are poor. The problems range from malnutrition, anaemia, communicable and non-communicable diseases, mental health, and substance abuse [9,10]. A study from Andhra Pradesh highlighted a higher prevalence of non-communicable diseases [11]. Kumar et al. (2020) reported poor health-seeking behaviour among the tribes owing to a lack of awareness, education, sociocultural beliefs, and socioeconomic factors [12]. Sinha et al. (2023) reported a higher prevalence of multiple morbidity conditions among the tribal geriatric population [13].

Senior citizens are a source of wisdom and guidance for families. In India, a person aged 60 years or above is considered elderly [3,4]. Older adults within these tribes hold a pivotal role, serving as custodians of cultural heritage and traditional knowledge. However, the tribal geriatric population face multiple vulnerabilities due to old age, belonging to the tribal community, being on the lower step of the socioeconomic ladder, etc. Multiple factors such as poverty, lack of access to knowledge, and limited access to healthcare services contribute to the health and health-seeking behaviour of the tribal elderly. In addition, frailty due to ageing, lack of income, and reliance on other members with poor socioeconomic conditions compound their vulnerability.

Universal health coverage (UHC) aims to ensure that all individuals and communities receive the necessary health services without suffering financial hardship. It encompasses various health services, including preventive, promotive, curative, rehabilitative, and palliative care. Tribal populations face various vulnerabilities, and their health is far below the general population. The life expectancy of the tribes (63.7 years) is far below that of the non-tribal population in India (67 years) [14,15]. Only 52% of the tribal children were fully vaccinated [16], compared to the national average of 67%, as reported by the Fifth National Family Health Survey (NFHS) [17]. Regarding maternal health, only 22% of the tribal women received adequate antenatal care [18] compared to the national average of 59.3% [17]. A study among the tribes in the present study area reported higher neonatal and infant mortality [19]. Under-five mortality is higher among the tribes than in the general population in India [5,19]. To add to the evidence further, 30% of the malaria cases are reported from tribals, who represent 8.6% of the population; the estimated prevalence of tuberculosis was 703 per 100,000 population compared to 256 in the general population [14,20]. In the context of UHC, it is important to assess the morbidity and health-seeking behaviour among vulnerable groups such as the tribal elderly to understand the existing scenario and identify the gaps in treatment-seeking behaviour. This study reports the morbidity, health-seeking behaviour, and related out-of-pocket expenditures (OOPEs) among the geriatric tribal population living in the Visakhapatnam district of Andhra Pradesh, India.

## Materials and methods

The present study was carried out in the Visakhapatnam district of Andhra Pradesh, India. Thirteen tribes, namely, Bagatha, Gadaba, Goudu, KondaKammara, Konda Dora, Khondh, Kotia, Mali, Nooka Dora, Porja, Valmiki, Dulia, and Mulia, including three PVTGs, namely, Gadaba, Khondh, and Porja, inhabit this area, constituting 14.4% of the district's population, which is 4.29 million according to the Census of India 2011 [1]. The present study is a part of an implementation research to improve healthcare access to tribal communities in the context of UHC. Elderly care is one component of comprehensive primary healthcare services. Through nationwide implementation research, we identified and addressed some of the barriers to implementing these services with quality and equity. A household survey following a multistage cluster random sampling method was conducted. Eleven tribal-dominant sub-districts were selected randomly, out of which four, namely, Dumbriguda, Hukumpeta, Gangaraju Madugula, and Pedabayalu, were included. Four primary health centre (PHC) areas, one each from the selected sub-district, were selected randomly. From each PHC area, a minimum of 10 villages were randomly selected, i.e., the PHC village, three sub-health centre villages, and a minimum of six villages with no health facility. Finally, a total of 58 villages/hamlets were included in the study. From each village, households were selected randomly from all directions and corners. Thus, a total of 2139 households were randomly selected from 58 villages. The details of sample size calculation are available elsewhere [16]. Trained interviewers collected the data from an adult member of the household. Data were incomplete for 36 households. Finally, 2103 households were included in the analysis. These 2103 households have 8880 members (4239 male and 4641 female), of which 598 were aged 60 years and above. This subset of data on these 598 elderly (60 years or above) was considered.

Data were collected on socio-demographic details, access to social welfare schemes, and illness experiences (episodic/acute illness, hospitalisation, chronic illness) in the past 12 months. Trained interviewers, using an interview schedule, collected data in the local language Telugu, on household socioeconomic details, illness experiences of the household members, treatment-seeking behaviour/source of treatment, and OOPEs (for the most recent acute illness and hospitalisation; the previous month’s OOPE for chronic illnesses). Acute illness is a sudden onset of symptoms and lasts for a short period, i.e., from a few days to a few weeks, whereas chronic illnesses develop slowly over time and may remain life-long. Hospitalisation is a minimum of one day stay in a health facility. Regarding calculating OOPEs, if the money spent was reimbursed through insurance or by the employer, it was deducted from the total expenditures to obtain OOPEs. The institute’s ethics committee of the corresponding author's institute approved the study protocol. All the study participants were informed about the study’s purpose, and their verbal consent was obtained before the interviews.

## Results

Table 1 presents the background characteristics. A total of 382 (63.9%) of the elderly were between 60 and 64 years old, and 94 (16%) were 70 years and above. A total of 430 elderly said they were the head of the household, and 128 (28.1%) were dependents. While 248 (41.5%) lost their spouses experiencing widowhood, 11 (1.8%) were separated from their spouse and two (0.3%) were never married. Regarding educational attainment, 502 (83.9%) had no formal schooling, 96 (16.1%) had a formal education, and only three (0.5%) completed graduation. Agriculture and agricultural labour were the primary occupations for 402 (67.2%) older adults, and 83 (13.9%) were not working presently. Regarding monthly income, 39 (6%) reported no personal income and 416 (69.6%) reported a monthly income of up to Indian rupee (INR) 5000. The average monthly income was INR 3783 (5% trimmed mean). Financial assistance in the form of an old age pension scheme (a social security scheme of financial assistance to the elderly below the poverty line referred to as the Indira Gandhi National Old Age Pension Scheme) was received by 409 (68.4%) of the present study elderly. The utilisation of the Mahatma Gandhi National Rural Employment Guarantee Act, which guarantees a minimum of 100 days of paid work to rural households, had been reported by 319 (17%), and 103 (17.2%) received financial assistance under the state government’s scheme Raitu Bharosa (meaning reassurance to farmers), under which a farmer gets an amount of INR 13500 in three spells in a year. It may be noted here that the government’s financial assistance in the form of old age pensions and other financial support contributed to the income.

**Table 1:** Sociodemographic characteristics and utilisation of government schemes. # Indira Gandhi National Old Age Pension Scheme provides financial assistance to people aged 60 years and above who are below the poverty line. * Mahatma Gandhi National Rural Employment Guarantee Act guarantees at least 100 days of wage employment in a financial year to rural households. $ Raitu Bharosa (financial support to the farmers): The government of Andhra Pradesh provided financial assistance of INR 13500 per year in three instalments to support the farmers for agricultural operations; 1 INR = US$ 0.012. INR: Indian rupee; SE: standard error.

Variable	Number (%)
Gender	
Men	317 (53.0)
Women	281 (47.0)
Age group (years)	
60-64	382 (63.9)
65-69	122 (20.4)
70 and above	94 (15.7)
Mean age ± SD	
Relation of the head of the household	
Self/head of the household	430 (71.9)
Dependent	168 (28.1)
Marital status	
Currently married	337 (56.4)
Never married	2 (0.3)
Widow/widower	248 (41.5)
Separated/deserted/divorced	11 (1.8)
Educational attainment	
No formal schooling	502 (83.9)
1-5 years of education	55 (9.2)
6-8 years of education	19 (3.2)
9-10 years of education	17 (2.8)
11-12 years of education	2 (.3)
15 or more years of education	3 (.5)
Primary occupation	
Agriculture/agricultural labour/daily wage labour	402 (67.2)
Self-employed/small business	5 (0.8)
Employed in private companies/non-governmental organisation	1 (0.2)
Retired employee	9 (1.5)
Homemaker	98 (16.4)
Not working currently	83 (13.9)
Income per month (including the old age pension)	
Did not have any personal income	39 (6.5)
Up to INR 5000	416 (69.6)
INR 5001-10000	100 (16.7)
More than INR 10000	43 (7.2)
Average monthly income ± SE	4398 ± 186
5% trimmed, mean ± SD	3783 ± 4560
Median	3000
Utilisation of government support systems	
Received financial assistance under the Indira Gandhi National Old Age Pension Scheme^#^	409 (68.4)
Utilised Mahatma Gandhi National Rural Employment Guarantee Act*	319 (53.3)
Received financial assistance under the farmer’s support programme, Raitu Bharosa^$^	103 (17.2)

Illness experiences over the past 12 months

Regarding illness experiences, 338 (56.8%) experienced some illness (either acute, chronic, or hospitalisation) in the past 12 months. While 277 (46.3%) reported experiencing at least one acute illness in the past 12 months, 224 (37.5%) had some chronic illness, and 17 (2.8%) were hospitalised (Table 2).

**Table 2:** Details of acute and chronic illness experiences and hospitalisation of the elderly. * Multiple illnesses were reported. CHC: community health centre; PHC: primary health centre; SHC: sub-health centre.

Illness details/variable	Number (%)
Number	598
Illness experiences	
Experienced acute/episodic illness in the past 12 months	277 (46.3)
Chronic illness present	224 (37.5)
Hospitalised in the past 12 months	17 (2.8)
Experienced any illness (acute, hospitalisation, chronic)	338 (56.8)
Number of acute/episodic illnesses	
No episodic illness in the past 12 months	321 (53.7)
Experienced at least one episodic acute illness in the past 12 months	277 (46.3)
Experienced one episode of illness	66 (11.0)
Experienced 2-3 episodes of illness	129 (21.6)
Experienced four or more episodes of illness	82 (13.7)
Acute illnesses experienced*	
No illness experienced	321 (53.7)
Body pains/musculoskeletal problems	170 (28.4)
Fever	71 (11.8)
Headache	37 (8.4)
Cough	28 (4.7)
Cold	20 (3.3)
Eye, ear, throat-related problems	13 (2.5)
Abdominal pain/digestive problems	13 (2.2)
Malaria	10 (1.7)
Weakness/nutrition-related problems	8 (1.3)
Swelling in body parts	6 (1.0)
Unintentional injuries (falls/burns/shocks)	3 (0.5)
Typhoid	2 (0.3)
Jaundice	1 (0.2)
Other (pain in chest/liver problem/skin problem/thyroid/urinary tract infections)	12 (2.0)
Source of treatment for the most recent episode (n = 277)	
Did not seek care/self-care/home remedies	14 (3.2)
Community health centre/primary health centre/sub-health centre	149 (53.8)
Area hospital/district/medical college hospital	50 (18.1)
Primary healthcare workers	25 (9.0)
Private hospital/nursing home/non-governmental organisation	22 (7.2)
Medical shop	10 (3.6)
Traditional healers/unqualified practitioners	7 (2.5)
Source of treatment	
Did not seek care/self-care/home remedies	14 (3.2)
Government facilities	226 (81.6)
Private facilities	37 (13.4)
Chronic illness	
No chronic illness	374 (62.5)
Chronic illness present	224 (37.5)
One chronic illness	195 (32.6)
More than one chronic illness	29 (4.5)
Chronic illnesses*	
Musculoskeletal problems	115 (19.2)
High blood pressure	75 12.5)
Diabetes	13 (2.2)
Paralysis	11 (1.8)
Eye-related problems	11 (1.8)
Chronic weakness	9 (1.5)
Respiratory/lung problems	5 (0.8)
Digestive diseases	3 (0.5)
Epilepsy	3 (0.5)
Tuberculosis	3 (0.5)
Heart-related problems	1 (0.2)
Kidney problem	1 (0.2)
Physical disability	1 (0.2)
Skin problems	2 (0.3)
Ill-defined conditions	2 (0.3)
Duration of chronic illness	
No chronic illness	374 (62.5)
For the last one year	55 (9.2)
2-3 years	106 (17.7)
4-5 years	16 (2.7)
6-10 years	31 (5.2)
For more than 10 years	16 (2.7)
Major source of treatment	
Not seeking treatment	109 (48.7)
PHC/SHC/CHC	54 (40.3)
Area hospital/district hospital	34 (27.4
Private hospital	22 (17.7)
Medical shop	3 (2.4)
From health workers/mobile medical unit	6 (4.8)
Type of provider	
No treatment/home remedies/self-medication	109 (48.7)
From government facility	94 (42.0)
Private facility	25 (11.2)
Hospitalisation	n (%)
Hospitalisation in the past 12 months	
No, not hospitalised	581 (97.2)
Yes, hospitalised	17 (2.8)
Reasons for hospitalisation	
Stroke	5 (29.4)
Abdominal pain	2 (11.8)
Motor vehicle accidents	2 (11.8)
Jaundice	2 (11.8)
Typhoid	2 (11.8)
Surgery	1 (5.9)
Arthritis/musculoskeletal problems	1 (5.9)
Kidney problems	1 (5.9)
Headache	1 (5.9)
Type of health facility admitted to	
PHC/CHC	3 (17.6)
Area hospital	4 (23.5)
District/medical college hospital	5 (29.4)
Private hospital	5 (29.4)

Acute illnesses

While 277 (46.3%) had at least one acute illness in the past 12 months, 129 (21.6%) experienced two to three acute illness episodes, and 82 (13.7%) experienced four or more episodes. Body pains were the most common problem for 170 (28%) elderly, followed by fever among 71 (11.8%) and headache among 37 (8.4%). Ten (1.7%) elderly had malaria. When asked about the treatment-seeking behaviour for the most recent episode of acute illness, 14 (3.2%) did not seek care or were limited to self-care. The major source of treatment was the primary healthcare system (Table 2). Health workers (auxiliary nurse-midwives (ANMs) and community health workers referred to as ASHAs - accredited social and health activists), who make regular visits in the community, were the source of treatment for 25 (9%) older adults, and seven (2.5%) reported to seek care from the traditional healers and unqualified practitioners (four from traditional healers and three from unqualified practitioners). Thus, the results reveal that a majority, i.e., 231 (83.4%), relied on government healthcare facilities for their healthcare needs.

Chronic illness

Chronic illness was present in 195 (37.5%) elderly persons. Musculoskeletal problems for more than one year were reported by 115 (19.2%), high blood pressure by 75 (12.5%), and diabetes by 13 (2.2%) elderly. Eleven (1.8%) had paralysis, 11 (1.8%) had eye-related problems, and respiratory problems were reported by five (0.8%). Of the elderly, 55 (9.2) reported they were having the reported chronic problems for one year, and 169 (28.3%) reported experiencing chronic illness for more than two years. Multimorbidity (the presence of two or more chronic conditions in a person simultaneously) was present in 29 (13.4%) of older adults. A majority, i.e., 92 (42.0%), relied on government health facilities, 25 (11.2%) relied on private healthcare facilities, and 109 (48.7%) were not seeking any care for their chronic conditions, mainly for musculoskeletal problems.

Hospitalisation

When asked whether they had been hospitalised in the past 12 months, 17 (2.8%) reported being admitted. While five (29.4%) were admitted due to stroke, two each (11.8%) were admitted for abdominal pain, motor vehicle accidents, jaundice, and typhoid. Twelve (70.6%) were admitted to government healthcare facilities (primary health centre/community health centre, area hospital, district hospital) and five (29.4%) were admitted to private hospitals.

Out-of-pocket expenditures

Table 3 presents the OOPEs of the elderly for acute illnesses. For acute illness, most, 152 (54.9%), spent INR 100 to 500 on the most recent acute illness episode. The average (5% trimmed mean) OOPE for the most recent acute illness was INR 387. These expenditures were higher when seeking care from private healthcare facilities (trimmed mean = 4301) than government health facilities (trimmed mean = 254).

**Table 3:** Out-of-pocket expenditures (OOPEs) for acute illnesses by source of care. 1 INR = US$ 0.012. INR: Indian rupee.

Expenditure	Did not seek care (n = 14)	Government healthcare facility (n = 226)	Private healthcare facility (n = 37)	Any (n = 277)
Did not spend	12 (85.7)	58 (25.7)	0 (0.0)	70 (25.3)
Up to INR 100	2 (14.3)	42 (18.6)	8 (21.6)	52 (18.8)
INR 101-200	0 (0.0)	55 (24.3)	4 (10.8)	59 (21.3)
INR 201-500	0 (0.0)	37 (16.4)	4 (10.8)	41 (14.8)
INR 501-700	0 (0.0)	5 (2.2)	0 (0.0)	5 (1.8)
INR 701-900	0 (0.0)	2 (0.9)	0 (0.0)	2 (0.7)
INR 901-1000	0 (0.0)	15 (6.6)	3 (8.1)	18 (6.5)
INR 1001-1500	0 (0.0)	2 (0.9	2 (5.4)	4 (1.4)
INR 1501-2000	0 (0.0)	2 (0.9	4 (10.8)	6 (2.2)
INR 2001-3000	0 (0.0)	3 (1.3)	1 (2.7)	4 (1.4)
INR 3001-4000	0 (0.0)	1 (0.4)	0 (0.0)	1 (0.4)
INR 4001-5000	0 (0.0)	3 (1.3)	4 (10.8)	7 (2.5)
INR 5001 & above	0 (0.0)	1 (0.4)	7 (18.9)	8 (2.9)
Mean ± SD	4 ± 3	423 ± 65.5	5763 ± 1728	1115 ± 259
5% trimmed mean	1.8	254	4301	387

The major source of money for acute illnesses (Table 4) was savings for 130 (46.9%), and 127 (45.8%) relied on their current income to meet their expenditures; seven elderly (2.5%) reported borrowing money from others or selling items for seeking treatment.

**Table 4:** Source of money for treatment of acute illnesses. * Multiple sources were reported.

Source of money	Number (%)*
Did not spend	70 (25.3)
Current income	127 (45.8)
Savings	130 (46.9)
Borrowed from others/sold items	7 (2.5)

Table 5 presents the OOPEs for chronic illnesses. The monthly average expenditure (5% trimmed mean) for chronic illnesses was INR 194. Seeking care from private facilities resulted in a trimmed average monthly expenditure of INR 3403 compared to that from government facilities (INR 262).

**Table 5:** Out-of-pocket expenditures (OOPEs) for chronic illnesses by source of care. 1 INR = US$ 0.012. INR: Indian rupee; OOP: out of pocket.

Monthly expenditure	Did not seek care (n = 112)	Government healthcare facility (n = 90)	Private healthcare facility (n = 22)	Any (n = 224)
Did not spend from OOP	107 (95.5)	18 (20.0)	0 (0.0)	125 (55.8)
Spent up to INR 500	4 (3.6)	44 (48.9)	3 (13.6)	51 (22.8))
INR 501-750	0 (0.0)	4 (4.4)	1 (4.5)	5 (2.2)
INR 751-800	0 (0.0)	1 (1.1)	0 (0.0)	1 (0.4)
INR 951-1000	0 (0.0)	3 (3.3)	1 (4.5)	4 (1.8)
More than INR 1000	1 (0.9)	20 (22.2)	17 (77.3)	38 (17.0)
Mean ± SD (in INR)	10 ± 77	1672 ± 10710	4155 ± 6354	1085 ± 7157
5% trimmed mean (in INR)	0.00	262	3403	194
Minimum (in INR)	0	0	0	0.00
Maximum (in INR)	800	100000	22000	100000.00

Regarding the source of money for chronic illness treatment (Table 6), 92 (41.1%) reported that current income was the source of money for seeking care for chronic illnesses, and 10 elderly reported borrowing money from others.

**Table 6:** Source of money for treatment of chronic illnesses. * Multiple sources were reported.

Source of money	Number (%)*
Current income/savings	92 (41.1)
Sold items	2 (0.9)
Contributed by relatives or friends	2 (0.9)
Borrowed from others	10 (4.5)
Treatment is provided free of cost	53 (23.7)

The hospitalisation expenditures (Table 7) ranged from INR 200 to INR 160000. The OOPE was higher when admitted to private hospitals (5% trimmed mean = INR 46063) than government facilities (5% trimmed mean = INR 6414). While 12 elderly (70.6%) reported savings as the source of money for hospitalisation, five (29.4%) reported borrowing money from others.

**Table 7:** Out-of-pocket expenditures (OOPEs) for hospitalisation by source of care. 1 INR = US$ 0.012. INR: Indian rupee.

Expenditures	Government hospital (n = 12)	Private hospital (n = 5)	Any hospital (n = 17)
Up to INR 500	4 (33.3)	0 (0.0)	4 (23.5)
INR 901-1000	2 (16.7)	0 (0.0)	2 (11.8)
INR 1001-2000	1 (8.3)	0 (0.0)	1 (5.90
INR 2001-4000	1 (8.3)	1 (20.0)	2 (11.8)
INR 8001-10000	1 (8.3)	1 (20.0)	2 (11.8)
INR 10001-15000	1 (8.3)	0 (0.0)	1 (5.9)
INR 15001-20000	1 (8.3)	0 (0.0)	1 (5.9)
More than INR 20000	1 (8.3)	3 (60.0)	4 (23.5)
Mean ± SD	8783 ± 17017	49610 ± 64281	20791 ± 39997
5% trimmed mean (in INR)	6414	46063	14201
Minimum (in INR)	200	3050	200
Maximum (in INR)	60000	160000	160000
Source of money			
Savings	8 (58.3)	4 (80.0)	12 (70.6)
Borrowed from others	4 (25.0)	1 (20.0)	5 (29.4)

## Discussion

The study highlights the vulnerability of the tribal geriatric population. In the present study, older adults were also characterised by low economic status and reliance on work involving strenuous physical labour in advanced age. It may be highlighted here that, according to the Census of India, while 20.5% of India’s population was below the poverty line, 40.5% of the Indian tribes lived below the poverty line [1]. While life expectancy at birth in India was 67 years, it was 63 years for the scheduled tribes [7]. Likewise, various health indicators among the tribes, except for the sex ratio, were far below the national average in India [14]. This highlights the vulnerability of the tribal populations. Another major stressor in older age is widowhood, characterised by a lack of companionship and support. Lower incomes further exacerbate this vulnerability. The study warrants strengthening the antipoverty measures, particularly social security, regarding old age pensions and access to free and quality healthcare. It may be noted that financial support from the government contributed to their income; otherwise, the average monthly income would have been much lower. Higher illiteracy, financial dependence, and stress were reported among the tribal elderly [21].

Some studies reported that widowhood among older adults is associated with declined quality of life [22] and mental health [23]. Zhu et al. identified intergenerational financial support as a partial mediator between widowhood and quality of life [22]. Economic, social, and psychological resources have been shown to mediate between widowhood and mental health partially [23]. However, in the present study context, their overall low socioeconomic status limits the chances of intergenerational financial support. Thus, financial, family and community support can improve the lives of the elderly, and support from the state remains crucial for this vulnerable section. Nonetheless, support from healthcare providers is crucial. In the background of the overall low socioeconomic status of the Indian tribes, ensuring social security, specifically in the form of old age pensions and improved access to quality healthcare services by the state, is crucial. Ashifa emphasised the need for pensions for the tribal elderly [24].

The present study reports a considerable illness burden among the tribal elderly, with more than 50% of the elderly experiencing some illness in the past 12 months. Chronic illnesses were considerable, and the major chronic illness among the present study's elderly was musculoskeletal problems for more than one year. A high burden of musculoskeletal problems can be due to their strenuous physical labour and hard geographical location with rough hilly terrains. Basu et al. reported a high prevalence of low back pain and joint pains among tribal elderly from the Indian state of West Bengal [21].

High blood pressure, diabetes, and multi-morbidity were considerable. A higher percentage (22.7%) of multi-morbidity among older adults in India was reported [13]. According to the Longitudinal Ageing Study in India, 32% of the elderly were hypertensive, and 14.2% had diabetes [25]. The present study reports a relatively lower prevalence of these conditions, which can be partially due to unawareness of the illness and lack of symptom recognition and diagnosis among this vulnerable population. This highlights the need for community awareness generation activities regarding health, illness symptoms, available healthcare services, and community-based health surveys.

It may be noted here that for hospitalisation and chronic illnesses, 20-30% of the people relied on private healthcare facilities. Given the average monthly incomes, the treatment expenditures were relatively high, mainly for hospitalisation and care for chronic illnesses. Higher OOPEs were reported for private healthcare facilities compared to government healthcare facilities [26]. It may be mentioned here that the transportation costs add not only to the OOPEs but also to the treatment delays. Public transport is grossly lacking in these areas, and people rely on private transport, mainly autorickshaws, which charge higher amounts due to limited service availability. Also, it is difficult for a sick person to walk longer distances to reach a health facility. It may be mentioned here that 50% of the villages are more than 1 km from the sub-health centre, and 80% are more than 2 km from PHC. It is very difficult for a sick person to walk long distances in rough hilly terrains, thus the lack of public transportation and road connectivity contributes to treatment delays and higher OOPEs. Hence, there is a need to improve access to healthcare and strengthen government healthcare services to meet the treatment needs of these vulnerable elderly. It is equally crucial to improve road connectivity so that all villages have all-weather roads and public transport is available at affordable cost. The study further reveals that the elderly mainly utilised their savings and borrowed money to meet the treatment expenditures. Thus, there is a need to ensure accessible, affordable, and acceptable healthcare to vulnerable populations.

Mavalankar highlighted the grossly underdeveloped healthcare services and lack of access to good quality services in the tribal areas in India [27]. Thus, addressing this situation by improving access to quality healthcare services by the government has been identified as crucial. In 2018, the government of India launched its flagship programme, Ayushman Bharat (meaning long live India), to achieve universal health coverage by 2030. Under this programme, improving access to comprehensive primary healthcare services is one component, and the other is financial protection in terms of health insurance by the state, which aims to cover the bottom 40% of the population in terms of socioeconomic status. To improve access to primary healthcare services, the sub-health centres that an auxiliary nurse midwife headed aimed at improving maternal and child health were being transformed into health and wellness centres (HWCs) with additional human resources, diagnostic facilities, and drugs. The HWCs are responsible for providing an expanded range of 12 comprehensive primary healthcare services, wellness activities, and appropriate forward and backward referrals. Elderly care is among the 12 comprehensive primary healthcare components through HWCs. The components of elderly and palliative care need to be strengthened with the necessary capacity-building of healthcare providers to cater to the specific needs of older adults. Strengthening the community partnerships through the existing village health, nutrition, and sanitation committees and Jan Aarogya Samiti (meaning people’s health forum) is important for ensuring appropriate and timely care for the elderly through the HWCs and mobile medical units in the present study district.

Regarding the study's limitations, its cross-sectional design limits causal inferences. It is carried out in only one district, which may limit the generalisability of the findings to other tribal populations that may differ in socio-economic and healthcare contexts. Self-reported illnesses may introduce recall bias. There is a possibility of inaccuracies or underreporting of OOPEs in the present context of limited financial literacy. Despite these possible limitations, the study’s strengths include focussing on a vulnerable and underrepresented tribal elderly population, contributing the existing knowledge and insights into their health and healthcare access. Although the sample is limited to one district, it used a large, diverse, and randomly selected sample, enhancing representativeness to the study’s setting.

Recommendations

The study emphasises the need to strengthen government healthcare services, particularly through HWCs under India’s Ayushman Bharat programme. Improving road connectivity with all-weather roads and public transportation facilities is equally crucial. Community awareness is also needed for symptom recognition and to improve health-seeking behaviour. Access to government social support schemes, such as old age pensions and health insurance, should be improved. Health education and community partnerships are to be strengthened. Addressing these gaps is vital to alleviating health disparities among the tribal elderly and securing their access to India’s universal health coverage framework.

## Conclusions

The illness burden is high among the tribal geriatric elderly. The majority relied on government healthcare facilities: 226 (81.6%) for acute illnesses, 94 (42%) for chronic illnesses, and 12 (70.6%) for hospitalisation. The OOPEs were high, particularly when seeking care from private health facilities. On average, acute and chronic illness expenditures consume nearly 30% of the monthly income. The OOPEs for hospitalisations were very high given the economic status of the present study elderly, and 29% of the elderly have to borrow money for hospitalisation.

Thus, the study warrants strengthening the poverty mitigation measures, improving access to healthcare services for the elderly through health and wellness centres, and strengthening community partnerships, especially in tribal areas.
